# Practitioners support and intention to adopt universal access to self‐collection in Australia's National Cervical Screening Program

**DOI:** 10.1002/cam4.7254

**Published:** 2024-05-24

**Authors:** Nicola Stephanie Creagh, Tessa Saunders, Julia Brotherton, Jane Hocking, Amalia Karahalios, Marion Saville, Megan Smith, Claire Nightingale

**Affiliations:** ^1^ Centre for Health Policy, Melbourne School of Population and Global Health The University of Melbourne Melbourne Victoria Australia; ^2^ Centre for Epidemiology and Biostatistics, Melbourne School of Population and Global Health The University of Melbourne Melbourne Victoria Australia; ^3^ Australian Centre for the Prevention of Cervical Cancer Carlton Victoria Australia; ^4^ The Daffodil Centre The University of Sydney, a joint venture with Cancer Council NSW Sydney New South Wales Australia

**Keywords:** cervical cancer, cervical screening, healthcare providers, implementation research, practitioners, qualitative, self‐collection, self‐sampling

## Abstract

**Objective:**

Primary care practitioners are crucial to engaging people in Australia's national cervical screening program. From July 2022, practitioners have been able to offer all screen‐eligible people the choice to collect their own self‐collected sample; an option introduced to increase equity. This study explored how practitioners are intending to incorporate universal access to self‐collection into their clinical care.

**Methods:**

Semi‐structed interviews with 27 general practitioners, nurses, and practice managers from 10 practices in Victoria, Australia conducted between May and August 2022. Interviews were deductively coded, informed by the Consolidated Framework for Implementation Research. The Diffusion of Innovations theory was used to categorise intention to provide self‐collection.

**Results:**

Participants were supportive of universal access to self‐collection, citing benefits for screen‐eligible people and that it overcame the limited adaptability of the previous policy. Most participants' practices (*n* = 7, 70%) had implemented or had plans to offer the option for self‐collection to all. Participants deliberating whether to provide universal access to self‐collection held concerns about the correct performance of the self‐test and the perceived loss of opportunity to perform a pelvic examination. Limited time to change practice‐level processes and competing demands within consultations were anticipated as implementation barriers.

**Conclusions:**

The extent to which self‐collection can promote equity within the program will be limited without wide‐spread adoption by practitioners. Communication and education that addresses concerns of practitioners, along with targeted implementation support, will be critical to ensuring that self‐collection can increase participation and Australia's progression towards elimination of cervical cancer.

## Graphical abstract

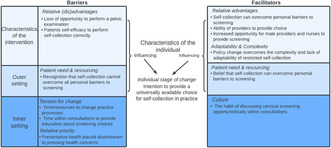

## 1 BACKGROUND

Australia could be one of the first countries to reach the World Health Organisation's threshold for the elimination of cervical cancer as a public health problem (≤4 cases per 100,000 women), projected to occur between 2028 and 2035.^1^, ^2^ Despite the current pace, without concerted efforts, Australia is at risk of leaving behind the very groups who currently bear the greatest cancer burden.^3^, ^4^, ^5^, ^6^, ^7^ Improving access and participation in cervical screening is essential to the equitable elimination of cervical cancer. Polymerase chain reaction (PCR) testing of samples obtained via vaginal self‐collection (hereafter referred to as self‐collection) for human papillomavirus (HPV) has equivalent sensitivity for the detection of CIN2+ compared to clinician‐collected samples, and has been demonstrated to both increase screening participation, and be highly acceptable to screen‐eligible people.^8^, ^9^, ^10^, ^11^, ^12^, ^13^ It should, therefore, contribute to more equitable outcomes in Australia's National Cervical Screening Program (NCSP).

In December 2017, when the switch from 2‐yearly Pap smears to 5‐yearly HPV tests occurred, guidelines restricted self‐collection to screen‐eligible people who were aged 30 years and older, were under‐ or never screened, and who declined a clinician‐collected sample (hereafter referred to as restricted access to self‐collection). This was due to concerns, at the time, about the marginally lower sensitivity to detect CIN2+, compared to clinician‐collected HPV screening.^14^ Under restricted access, the uptake of self‐collection was very low, with only 0.1% (*n* < 6000) of all screening tests within the first 2 years being self‐collected, representing less than 1% of more than one million screen‐eligible people estimated to have been eligible.^15^, ^16^ Subsequently, Australia updated the cervical screening policy, based on updated evidence, to allow everyone due for screening the choice to be screened using either a self‐collected or clinician‐collected sample (hereafter referred to as universal access to self‐collection).^11^, ^17^, ^18^ The policy change took effect on July 1, 2022.

In Australia, cervical screening is typically provided through primary care, often opportunistically within consultations, but is also facilitated by practice‐based and centralised registry‐based reminders and invitations. Ordering screening tests and accessing reimbursement from the government's universal health insurance scheme requires a Medicare Benefits Scheme provider number, thus largely restricting the provision of cervical screening to general practitioners (GP) and specifically trained nurse practitioners. Access to self‐collection is facilitated through this model (hereafter referred to as a practitioner‐supported delivery model). In practice, this means that the test and instructions are provided in a clinical setting and discussed during a consultation, including telehealth consultations or settings that the supervising practitioner believes to be suitable.^17^ Participants opting for self‐collection who are positive for the most oncogenic HPV types (16/18) are referred directly to colposcopy, whereas those who are HPV+ (non 16/18) are invited back to the GP for the collection of a cervical sample for cytology. The provision of self‐collection through a practitioner‐supported delivery model maintains the critical role of primary care providers in supporting the delivery of screening and follow‐up care. It also means that the availability of self‐collection is limited to patients of practitioners who adopt and offer self‐collection in practice.

In the absence of direct evidence on the extent to which primary care practitioners in Australia had adopted and delivered restricted access to self‐collection, its substantially low uptake suggests that its integration within primary care was low.^15^, ^16^ Several barriers to the provision of restricted access to self‐collection within primary care have been reported, including limited awareness, varied beliefs about its suitability and accuracy, the need to have timely access to screening histories to determine eligibility, and varied access to pathology laboratories accredited to test samples.^8^, ^19^, ^20^, ^21^, ^22^, ^23^, ^24^, ^25^ The challenge of needing to confirm eligibility has been largely overcome through both simplified guidelines allowing universal access to self‐collection, and improved access to the National Cancer Screening Register.^26^ However, practitioners' concerns about the suitability and accuracy of self‐collection and their access to pathology support may persist. If so, this will not only impact the implementation of universal access to self‐collection within Australia's health system but also its capacity to improve screening participation and equity within the program.

Given the use of a practitioner‐supported delivery model, the key challenge to the effective implementation for self‐collection in Australia is to ensure practitioners adopt and deliver universal access to self‐collection. Pre‐implementation readiness, defined by Holt et al (2010) as the extent to which practitioners and their structural setting is primed, motivated, and capable of executing a change, is an important precursor to the implementation of an innovation, such as universal access to self‐collection.^27^ This study therefore aimed to describe how practitioners and people working within primary care perceived universal access to self‐collection in the immediate lead‐up to and directly after its introduction within national policy. We further explored their intention to provide universal access to self‐collection in practice, and the anticipated or experienced barriers to doing so.

## 2 METHODS

This study employed a qualitative approach, utilising semi‐structured one‐on‐one or group interviews with professionals (GPs, nurses, practice managers) working in primary care sites in Victoria, Australia.

### 2.1 Theoretical approach

The Consolidated Framework for Implementation Research (CFIR) Version 1 was used to underpin data collection and analysis for this study. The CFIR comprises five domains and 39 constructs and has been used in qualitative analyses exploring contextual factors (i.e., barriers or facilitators) to influence implementation within healthcare settings.^28^ With the focus of this study being on the early perception and experience of healthcare professionals, the CFIR domains of most relevance include, (1) characteristics of the intervention, (2) the inner setting, (3) the outer setting; and (4) the characteristics of the individuals. In addition, the *Innovation‐Decision* process of the Diffusion of Innovations theory was used to categorise the *“individual stage of change”* construct of CFIR's *“*characteristics of individual's*”* domain.^29^

### 2.2 Recruitment

Primary care sites in Victoria, Australia were recruited in two ways: (1) advertisment through Primary Health Networks, or (2) Australian Centre for the Prevention of Cervical Cancer delivered practitioner education sessions. Eligible primary care sites were those who provide cervical screening as part of their clinical care and had at least one or more professionals (GP, nurse, practice manager) who provided consent to be involved in the study. All participants were reimbursed for their involvement, through a gift card or invoice at AUD150.00 per hour.

### 2.3 Data collection

One‐on‐one or didactic semi‐structured interviews were conducted in person at the practice, by telephone, or by zoom, by a university‐level trained qualitative researcher (N.S.C., PhD Candidate, female). Group interviews were conducted with two or more members from the same practice, this was at their request. An interview guide was developed by the authorship team and covered themes including (a) practice culture and processes around the delivery of cervical screening, (b) perception and previous experience with providing restricted access to self‐collection; and (c) perception of and intention to provide universal access to self‐collection. Interviews were conducted between May and August 2022; which aligned with the timing of policy change to universal access to self‐collection (July 1, 2022).

### 2.4 Data analysis

Interviews were audio recorded and later transcribed verbatim by an automated transcript service (Otter.ai). Prior to analysis, transcripts were de‐identified and cross‐checked against the recording to ensure data accuracy. Participants were provided the choice to receive and provide feedback on the transcript from their interview. Analysis was performed by deductive coding based on the constructs of CFIR in NVivo (release 1.6.2., QSR International Pty Ltd). Complete coding of the data was conducted by the first author (N.S.C.). To ensure interrater reliability, two transcripts (15%) were double coded by a second coder (T.S.), with conflicts discussed in‐depth by coders until agreement was reached. Double coding of transcripts ceased after high concordance was reached. After analysis, participants' reported intention to provide universal access to self‐collection (individual stage of change) was categorised to the Innovation‐Decision process of the Diffusion of Innovations theory^29^ by the first author (N.S.C.).

### 2.5 Ethics approval

This study obtained ethical approval through the University of Melbourne, Human Ethics Committee (2022–23, 089–31, 430‐7). All participants provided informed written or verbal consent (after hearing a consent script) prior to their involvement in the study.

## 3 RESULTS

**FIGURE 1:**
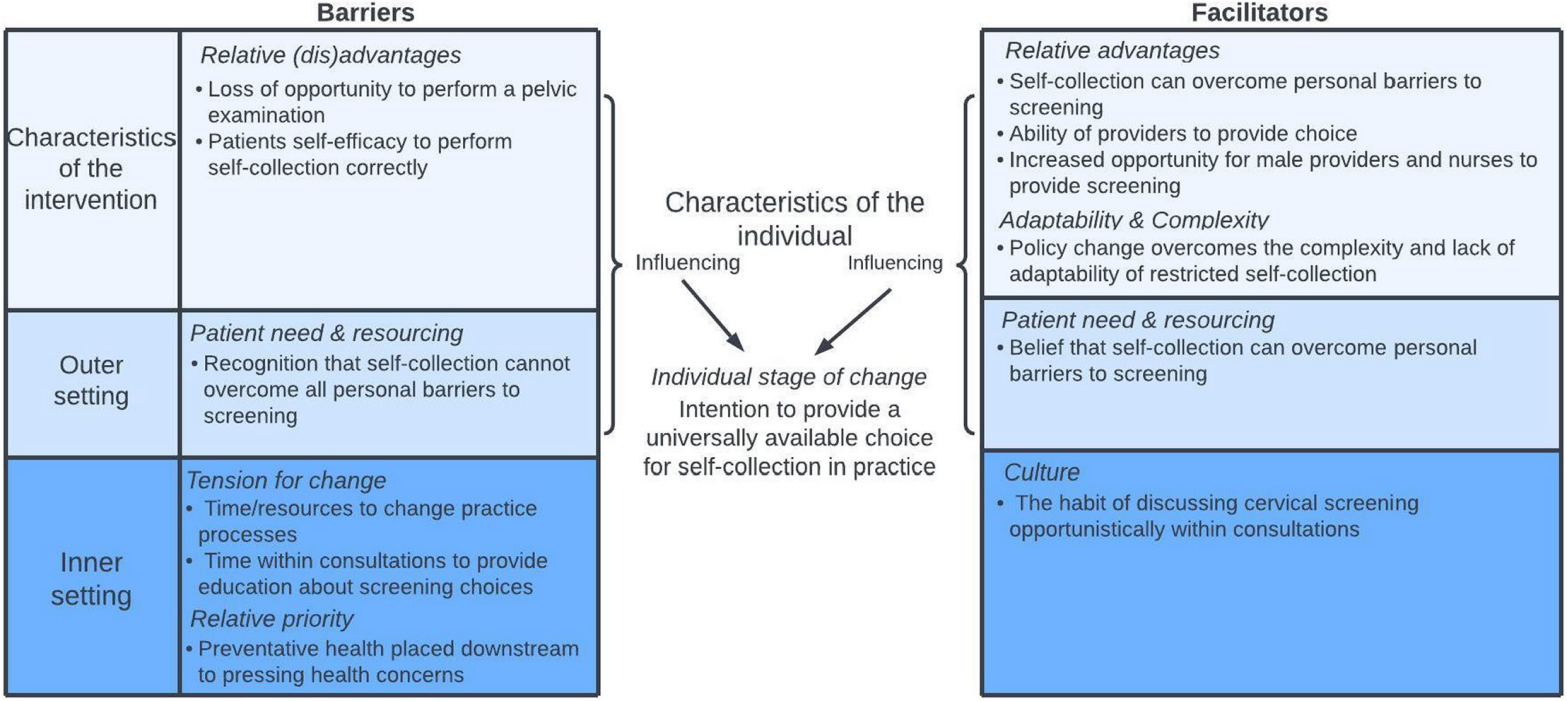
An overview of the major themes related to the CFIR domains and constructs pertaining to the implementation of universal access to self‐collection cervical screening from semi‐structured interviews of 27 practitioners and practice managers from 10 primary care sites, Victoria, Australia 2022.

A total of 27 participants were interviewed, from 10 primary care sites in Victoria Australia. Interviews with single participants (*n* = 7) lasted an average of 44 min and didactic (*n* = 3) lasted on average 47 min. Of these participants, 18 (67%; metropolitan area = 14 (78%), rural area = 4 (22%)) were GPs, 7 (26%; metropolitan area = 3 (43%), rural area = 4 (57%)) were nurses and 7% (*n* = 2; metropolitan area = 1 (50%), rural area 1 = (50%)) were practice managers. Most participants were female (*n* = 19; 70%), including all nurses and practice managers. All GPs and 3 of the 7 (43%) nurses provided cervical screening as part of their clinical care. The remaining nurses (57%, *n* = 4), and practice managers supported the delivery of screening within their practice (i.e., responsible for the practice's quality improvement activities and the administration of recalls). Just under one half of participants (11/27, 41%) were interviewed prior to policy change (May and June 2022), with the remaining 16 (59%) interviewed shortly after policy change (July to August 2022). The key themes surrounding the CFIR domains are presented below and summarised in Figure 1.

### 3.1 Characteristics of the intervention (self‐collection), defined as key characteristics of the innovation itself^28^

Participants were supportive of the policy change which made self‐collection universally available as a choice for all screening participants. This support was influenced by their reflection of several *relative advantages*, including the perception that self‐collection can overcome personal barriers experienced by screen‐eligible people to clinician‐collected screening:

*I thought it [self‐collection] was fantastic, because a lot of women hate coming in and having the speculum put in*. Practice 2, nurse, metropolitan area.

Further advantages included the ability for practitioners to provide options to their patients for how they can perform cervical screening, and the potential for an increased role for male practitioners and practice nurses in the delivery of cervical screening:

*I think it's about having a choice, isn't it? … I think if someone is hesitating, they can have a choice on what they can do*. Practice 1, GP, metropolitan area.

Policy change providing universal access to self‐collection was considered to largely overcome the *complexity* and limited *adaptability* which had been experienced when access to self‐collection was restricted.

*Initially I kind of go ‘[with restricted access to] self‐collection, you know, that's going to open up so much more for so many people’. But then when you read the eligibility and the guidelines [for restricted access to self‐collection], it was like, actually, maybe not. … In fact, I don't think I ever did one*. Practice 8, nurse, rural area.

There were, however, several *relative disadvantages* discussed by participants. These concerns centred around patient's self‐efficacy to perform self‐collection and the perception of the loss of opportunity to perform a pelvic examination, which may identify incidental health issues:

*My big concern has always been, is it going to be an adequate sample? Because a patient's doing it, whereas we have our own visualisation. So that's my main concern of self‐collection*—Practice 6, GP, metropolitan area.

*I still maintain that I think it's great that we have another alternative to the traditional smear test and collection. It was just, though, that it doesn't replace the examination which can pick up all of the other incidentary [sic] findings that you might get through a more thorough examination—*Practice 9, nurse, rural area.

### 3.2 Characteristics of the individual, defined as individuals within the setting that the innovation is implemented in^28^

**FIGURE 2:**
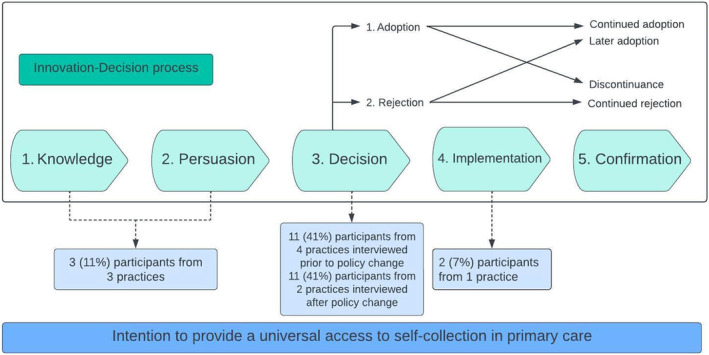
Participants reported intention to provide universal access to self‐collection cervical screening, mapped to the Diffusion of Innovation innovation‐decision process. The boxed component of the figure is adapted from Rogers (2003)^29^ and describes the innovation‐decision process, (1) *Knowledge*: where one is exposed to an innovation's existence, (2) *Persuasion*: where one forms a favourable and/or unfavourable attitude towards the innovation, (3) *Decision*: where one engages in activities that lead to a choice to adopt or reject the innovation, (4) *Implementation*: where one puts a new idea into use, (5) *Confirmation*: where one seeks reinforcement of an innovation‐decision already made.

Mapping participant's intention to provide universal access to self‐collection along the Diffusion of Innovation's Innovation‐decision process highlighted the difference in reported intention to provide the option for self‐collection (Figure 2).

Two participants (7%) from one practice (10%), interviewed 1 month after policy change, reported having fully implemented the guidelines, offering all eligible patients a choice for self‐collection. These participants also reported implementing pro‐active strategies in the provision of information about self‐collection to screen‐eligible people:

*What we will do now, and I know we started it as of the first of July [sic], so for any woman who's in the practice and comes in and books an appointment on‐site for cervical screening, reception staff will give them a letter [with information about self‐collection]. … If they've booked the appointment online, again, the girls [reception staff] are going back just to double check what appointments have been booked, and then they will email them that information about self‐collection to say it's an option*—Practice 8, nurse, rural area.

Participants (*n* = 22, 81%) from six practices (60%) reported a decision to adopt universal access to self‐collection, but were at varying stages of implementation, noting that some (*n* = 11, four practices) were interviewed prior to July 1, 2022 (policy change). The *relative advantages* for self‐collection were key influences in their decision to provide a choice for self‐collection to all screen‐eligible people:

“*My thoughts are that if self‐collection is just as sensitive as a practitioner cervical screening test, we should be offering it [self‐collection] to everyone*.”—Practice 4, GP, rural area.

Participants (*n* = 3, 11%) from the remaining three practices (30%) were within the knowledge and persuasion stages of the *Innovation‐Decision* process and unsure whether universal access to self‐collection would be fully adopted within their practice. At one practice, the practice manager indicated that while they themself were supportive of self‐collection, this may not change practitioner behaviour:

*I don't think the doctors are going to change any approach. … Now that of course it's [self‐collection] is available for everyone, we're [practice‐support staff] going to create definitely awareness. … But I don't think their [GPs] approach is going to change because they will still do what they are doing*—Practice 7, practice manager, rural area.

The *relative disadvantages* and practitioner's understanding (self‐efficacy) of the clinical practice guidelines were key considerations which influenced their indecisiveness on whether to provide the option for self‐collection universally.

### 3.3 Outer setting, defined as the economic, political, and social context within which the setting that the innovation is implemented resides^28^

Aligned with participant's perception of the *relative advantages*, *patient* (i.e., screen‐eligible people) *needs and resources* were key facilitators in participant's considerations around the implementation of universal access to self‐collection within their practice.

*I've offered it to a few women that I thought were physically a bit disabled, so they're like really obese or got terrible arthritis and too hard to get up on the couch. So they've taken the opportunity because they haven't been able to do it previously*. Practice 4, GP, rural area.

Participants, however, conveyed that the expanded availability for self‐collection would not address all barriers experienced by screen‐eligible people, particularly those related to a lack of understanding of the importance of screening and the aversion to discussing female issues with a male practitioner.

*I think there is some people that are averse to someone else collecting it, and some people averse to keep it to themselves as well. … Sometimes that doesn't resolve the issue, offering self‐collection—*Practice 1, GP, metropolitan area.

### 3.4 Inner setting, defined as the setting in which the innovation is implemented^28^

It was evident that participants and their practices maintained a strong *culture* in the provision of cervical screening, with several participants reporting that they maintain a habit of discussing screening opportunistically within consultations. Among participants who reported a decision to provide universal access to self‐collection, key barriers related to the *implementation climate* of primary care were reported. Implementation challenges including a lack of time and human resources to be able to implement new innovations effectively were cited, thus creating a *tension for change*:

*The barrier is having the time in the first place to make sure you understand what you're doing and setting it up and utilising it—*Practice 2, nurse, metropolitan area.

Participants also reflected on the limited time within consultations to provide the necessary education about their options between self‐collection or clinician‐collection.

*I was thinking… “Oh, great, it'll [providing self‐collection] be quicker” you know, and won't have to do speculums, but you're going to actually spend quite a bit of time explaining the difference and the options, so I don't think it'll be any quicker*—Practice 4, GP, rural area.

The issue of time further extended to the *relative priority* of cervical screening within consultations, noting that the priority of preventative health can be placed downstream to acute health concerns with which screen‐eligible people present to primary care:

*I think a lot of patients who have, maybe if they have quite complicated histories, or otherwise have other chronic conditions, I think often it [cervical screening] just gets… there's always so much to get through in a consultation, that that often gets missed*—Practice 6, GP, metropolitan area.

## 4 DISCUSSION

To our knowledge, this is the first description of the pre‐ and early implementation readiness of universal access to self‐collection within primary care in Australia. Participants were supportive of the policy change that expanded access to self‐collection, citing benefits for screen‐eligible people who experience barriers to clinician‐collected screening and that it largely overcomes the complexity of the provision of restricted access to self‐collection. Further, the wider availability of self‐collection was seen to provide greater opportunities for male providers and practice nurses to be involved in the provision of cervical screening. However, participants from only 70% (*n* = 7/10) of practices reported that they had adopted, or had plans to adopt, universal access to self‐collection into routine care. Concerns about the ability of screen‐eligible people to perform self‐collection and the perceived loss of opportunity to perform a pelvic examination were key considerations of participants deliberating their intention to provide universal access to self‐collection. Implementation barriers, including limited time to change practice level processes and competing demands within consultations, were also anticipated.

The implementation climate of universal access to self‐collection in Australia is challenging because it comes directly after a policy setting that led to major implementation challenges, including practitioners lingering perceptions that self‐collection is less suitable and accurate.^15^, ^16^, ^19^, ^20^, ^21^ While participants reflected on the benefit of the practitioner‐supported model of care, the fact that some practitioners have not yet decided to adopt the provision of an option for self‐collection will have considerable implications for its availability. Favourable attitudes towards an innovation influences the likelihood of making a decision to adopt the innovation,^29^ and certainly practitioners in our study who held fewer concerns about self‐collection were more likely to report a decision to adopt universal access to self‐collection. Further work is required to fully understand, at a national level, the workforce's support for, and intention to provide, the option of self‐collection when they offer cervical screening.

Of note was the prevalent concern among practitioners in this study that screen‐eligible people may not correctly collect their own samples. While there are studies that indicate screen‐eligible people have concerns about how to collect a self‐collected sample, these were largely in studies among people who had not yet tried self‐collection.^31^, ^32^ Investigations among screen‐eligible people who have used self‐collection commonly report participants describing the test as easy and comfortable.^10^, ^12^ Further, data from the first 2 years of the self‐collection in Australia shows that only 2% of self‐collection tests yielded unsatisfactory laboratory results, similar to unsatisfactory rates for cervical cytology, but still higher than the invalid rate of clinician‐collected samples (0.3%).^8^, ^16^ Invalid results can be due to inhibition of the PCR reaction due to the presence of blood or other substances or a lack of cellular material.^33^ While the PCR assays do not distinguish between these causes, inhibition would be expected to affect both clinician and self‐collected samples. The difference in invalid rates between clinician and self‐collection is likely driven by a failure to sample the vagina when the patient does not want to refuse the offer for self‐collection, but is also not comfortable to collect their own sample.^33^ It is further important to reinforce that tests on self‐collected samples are assessing the presence of HPV‐DNA shed to the vagina, rather than assessing cervical cells. The fact that this concern was prevalent among practitioners speaks to the importance of ongoing education for practitioners on the relatively low unsatisfactory self‐collection rate and the reasons, beyond patients' ability to perform the test, which may lead a sample to be unsatisfactory.

The perceived loss of opportunity to perform a pelvic examination was another key concern reported within this study. The clinical practice guidelines state that “routine inspection of the cervix, vagina and external genitalia is not necessary or indicated for asymptomatic people,”^17^ making a clear delineation between pelvic examination on the basis of symptoms rather than as an examination for asymptomatic people. While clinicians concerns was acknowledged in a global evidence review surrounding routine pelvic examinations, it ultimately concluded that there was no direct evidence surrounding its benefit or harms.^34^ Likewise, both the Canadian Task Force on Preventive Health Care and the American College of Physicians, after reviewing the evidence, recommended against routine pelvic examinations in the absence of symptoms.^35^, ^36^ Certainly, any such examination requires transparent discussion and informed patient consent. Current evidence therefore substantiates Australia's guidelines; however, the clinical practice of practitioners is informed not only by classical evidence, but also by conversations with colleagues and personal clinical experience.^37^ The strength of recommendations within clinical guidelines, isolated from the other experiences that influence clinical judgements, may not be enough to overcome some practitioners' belief that routine inspection of the cervix, vagina and external genitalia is ideal in asymptomatic people, and thus hold the belief that self‐collection is an inferior clinical test.

We note that while practitioners involved in this study did not particularly raise concerns surrounding a lack of follow‐up after a positive HPV result, other studies conducted outside of the Australian context have.^46^, ^48^ It is, thus, critical that any increase in screening participation due to the availability of universal access to self‐collection is accompanied by high follow‐up rates for those who test positive for HPV. Available data demonstrates that in 2022, 76.7% of participants who tested HPV positive (16/18) after a self‐collected test had a colposcopy within 6 months; marginally lower than that the 6‐monthly follow‐up rate to colposcopy for the overall program (78.6%).^30^ In the same period, 77.4% of participants who tested HPV+ (non 16/18) after a self‐collected test has a repeat clinician‐collected test for a reflex liquid‐based cytology typically performed by an additional primary care visit, as recommended within the guidelines.^30^ It is plausible that those who experienced increased barriers to clinician‐collected screening could also experience barriers to follow‐up. Further work should monitor real‐world trends in follow‐up and implement appropriate health‐system strategies to support those through the screening pathway.

The spread, scale‐up and sustainability of change within healthcare systems has been described as a process that spans a continuum, in which often unpredictable dynamics, contextual factors and organisational processes can facilitate or impede progress.^38^ Anticipated barriers reported within this study included a tension to change, created by a lack of time and resources, and a relatively lower priority of screening compared to acute health issues may impact progress towards the spread and sustainability universal access to self‐collection in practice. An implementation science approach contends that the implementation of innovations can be achieved through a structured approach, where implementation supports are developed, replicated and evaluated to address dynamics, contextual factors and organisational processes.^39^ A plethora of tools and materials have been developed by stakeholders, including the National Cervical Screening Program. These include education modules and resources for practitioners, and information guides and instructions for screen‐eligible people. The extent to which these resources are reaching practitioners and screen‐eligible people, however, is not yet understood. Nor have they been evaluated to determine their effectiveness. This should be the focus of future work.

While this study is focused on the Australian primary care context, our findings will be relevant to the international community. As of February 2021, Australia was one of 17 countries with an organised cervical screening program to implement self‐collection as a primary or alternative screening modality.^40^ Aotearoa New Zealand is planning their transition to HPV‐based testing in July 2023, with a universal option for self‐collection delivered in a practitioner‐supported model. Furthermore, trials conducted in the United Kingdom on self‐collection have also used a practitioner‐supported model of care.^41^ Global systematic reviews demonstrate the effectiveness of self‐collection within mail‐out and door‐to‐door delivery models, noting that the contexts in which these are trialled vary substantially.^10^, ^11^, ^13^ Research on practitioner‐supported models is beginning to emerge^42^, ^43^, ^44^, ^45^, ^46^, ^47^, ^48^; however, these studies were all formative explorative studies or were in a trial setting. A key strength of our study is that data collection was conducted, in a real‐world setting, either directly before or after a policy change that allows universal access to self‐collection. This allowed us to outline practice implications, highlighting important learnings that may be applicable to other contexts.

### 4.1 Clinical implications

The introduction of universal access to self‐collection in Australia is well supported by evidence showing that it can increase participation and is acceptable to screen‐eligible people who decline clinician‐collected cervical screening.^10^, ^11^, ^12^, ^13^ Under the Australian clinical guidelines, all screen‐eligible people should be provided the choice to self‐collect their own sample.^17^ However, findings from our study demonstrate that this choice may not consistently be provided by practitioners. Frequent and consistent communication and education to the primary care workforce, addressing concerns identified in this study, highlighting (a) that self‐collected samples are just as accurate as clinician collected samples for the detection of CIN2+ (b) that most self‐collected samples yield satisfactory laboratory results, indicating adequate sample collection; and (c) that routine pelvic examinations for asymptomatic screen‐eligible people are not clinically necessary, can help to dispel key concerns of practitioners around self‐collection.

### 4.2 Study limitations

Our study provides valuable insights about universal access to self‐collection from the perspective of people working within primary care, at the time the policy was introduced in Australia. However, with a limited sample size from only one jurisdiction (Victoria), we acknowledge that there are likely to be diverse perceptions not captured. Through stakeholder networks, anecdotally, we understand that there are practitioners who are not supportive of universal access to self‐collection and this study did not include their perspectives. Further, a recent survey highlighted a higher acceptability for self‐collection from Victorian practitioners, compared to practitioners in other jurisdictions.^22^ Nevertheless, despite this limitation, our findings are likely to have important implications and learnings to guide the continuing roll‐out of universal access to self‐collection in Australia.

## 5 CONCLUSION

Universal access to self‐collection within Australia's National Cervical Screening Program is an essential part of Australia's national strategy to achieve equitable and timely elimination of cervical cancer as a public health problem.^49^ Our study demonstrates that, despite overarching support from practitioners and other people working in primary care for self‐collection and its universal availability, implementation challenges remain due to practitioner concerns, limited time to adapt practice‐level processes and competing priorities in consultations. Without wide‐spread adoption by practitioners, the full potential for self‐collection to increase screening participation and promote equity within the program will not be realised. Continued and consistent effort to promote self‐collection to practitioners in a way that addresses key concerns will be critical. Likewise, it is imperative that implementation strategies are developed, replicated and evaluated to support the implementation of a universal access to self‐collection within primary care in Australia.

## AUTHOR CONTRIBUTIONS

**Nicola Stephanie Creagh:** Data curation (lead); formal analysis (lead); investigation (lead); project administration (lead); writing – original draft (lead); writing – review and editing (lead). **Tessa Saunders:** Formal analysis (supporting); writing – original draft (supporting); writing – review and editing (supporting). **Julia Brotherton:** Conceptualization (equal); investigation (equal); methodology (equal); supervision (supporting); writing – original draft (supporting); writing – review and editing (supporting). **Jane Hocking:** Conceptualization (supporting); investigation (supporting); methodology (supporting); supervision (supporting); writing – original draft (supporting); writing – review and editing (supporting). **Amalia Karahalios:** Conceptualization (supporting); investigation (supporting); methodology (supporting); supervision (supporting); writing – original draft (supporting); writing – review and editing (supporting). **Marion Saville:** Investigation (supporting); methodology (supporting); writing – original draft (supporting); writing – review and editing (supporting). **Megan Smith:** Investigation (supporting); methodology (supporting); writing – original draft (supporting); writing – review and editing (supporting). **Claire Nightingale:** Conceptualization (equal); formal analysis (supporting); funding acquisition (equal); investigation (equal); methodology (equal); supervision (lead); writing – original draft (equal); writing – review and editing (equal).

## CONFLICT OF INTEREST STATEMENT

MS is an investigator on the Compass trial for which her organisation, The Australian Centre for the Prevention of Cervical Cancer (ACPCC), has received kits and partial funding from Roche. ACPCC/VCS Pathology has received equipment or supplies from Abbott, AusDiagnostics, BD, Cepheid, Copan, Hologic, Microbiologics, MicroBix, NRL, Qiagen, Rovers, Roche, and Seegene for research and validation studies. JMLB was previously employed by the Australian Centre for the Prevention of Cervical Cancer (ACPCC). ACPCC/VCS Pathology has received equipment or supplies from Abbott, AusDiagnostics, BD, Cepheid, Copan, Hologic, Microbiologics, MicroBix, NRL, Qiagen, Rovers, Roche, and Seegene for research and validation studies.

## ETHICS STATEMENT

This study obtained ethical approval through the University of Melbourne, Human Ethics Committee (2022–23,089–31,430‐7). All methods were carried out in accordance with relevant guidelines and regulations. All participants provided informed written or verbal consent (after hearing a consent script) prior to their involvement in the study.

## Supporting information


Data S1.

## Data Availability

Datasets used and/or analysed during the current study are available from the corresponding author on reasonable request.

## References

[1] Hall MT , Simms KT , Lew JB , et al. The projected timeframe until cervical cancer elimination in Australia: a modelling study. Lancet Public Health. 2019;4(1):e19‐e27. 30291040 10.1016/S2468-2667(18)30183-X

[2] World Health Organisation Global Strategy to Accelerate the Elimination of Cervical Cancer as a Public Health Problem. World Health Organisation; 2020.

[3] NHMRC Centre of Research Excellence in Cervical Cancer Control . Cervical Cancer Elimination Progress Report: Australia's Progress towards the Elimination of Cervical Cancer as a Public Health Problem. NHMRC C4; 2023. Available from: https://report.cervicalcancercontrol.org.au/

[4] Kerr L , Fisher CM , Jones T . Improving cervical screening in trans and gender‐diverse people. Cancer Nurs. 2022;45(1):37‐42. 32976182 10.1097/NCC.0000000000000890

[5] Curmi C , Peters K , Salamonson Y . Barriers to cervical cancer screening experienced by lesbian women: a qualitative study. J Clin Nurs. 2016;25(23–24):3643‐3651. doi:10.1111/jocn.12947 26264131

[6] Yu XQ , Feletto E , Smith MA , Yuill S , Baade PD . Cancer incidence in migrants in Australia: patterns of three infection‐related cancers. Cancer Epidemiol Biomarkers Prev. 2022;31(7):1394‐1401. 35322272 10.1158/1055-9965.EPI-21-1349 PMC9306400

[7] Whop LJ , Garvey G , Baade P , et al. The first comprehensive report on indigenous Australian women's inequalities in cervical screening: a retrospective registry cohort study in Queensland, Australia (2000‐2011). Cancer. 2016;122:1560‐1569. 27149550 10.1002/cncr.29954 PMC5074237

[8] Creagh NS , Zammit C , Brotherton JM , et al. Self‐collection cervical screening in the renewed National Cervical Screening Program: a qualitative study. Med J Aust. 2021;215(8):354‐358. 34145591 10.5694/mja2.51137

[9] Creagh NS , Zammit C , Brotherton J , et al. The experience of under‐screened and never‐screened participants using clinician‐supported self‐collection cervical screening within the Australian National Cervical Screening Program. Women's. Health. 2022;18:18. 10.1177/17455065221075905 PMC8841921 35147064

[10] Creagh NS , Boyd LAP , Bavor C , et al. Self‐collection cervical screening in the Asia‐Pacific region: a scoping review of implementation evidence. JCO Glob Oncol. 2003;9:e2200297. 10.1200/GO.22.00297 PMC10166429 36724416

[11] Arbyn M , Smith SB , Temin S , Sultana F , Castle P , Collaboration Self‐Sampling HPVT . Detecting cervical precancer and reaching underscreened women by using HPV testing on self samples: updated meta‐analyses. BMJ. 2018;363:k4823. 30518635 10.1136/bmj.k4823 PMC6278587

[12] Camara H , Zhang Y , Lafferty L , Vallely A , Guy R , Kelly‐Hanku A . Self‐collection for HPV‐based cervical screening: a qualitative evidence meta‐synthesis. BMC Public Health. 2021;21(1):1503. 34348689 10.1186/s12889-021-11554-6 PMC8336264

[13] Costa S , Verberckmoes B , Castle PE , Arbyn M . Offering HPV self‐sampling kits: an updated meta‐analysis of the effectiveness of strategies to increase participation in cervical cancer screening. Br J Cancer. 2023;128(5):805‐813. 36517552 10.1038/s41416-022-02094-w PMC9977737

[14] Arbyn M , Verdoodt F , Snijders PJF , et al. Accuracy of human papillomavirus testing on self‐collected versus clinician‐collected samples: a meta‐analysis. Lancet Oncol. 2014;15(2):172‐183. 24433684 10.1016/S1470-2045(13)70570-9

[15] Smith M , Saville M , Canfell K . HPV swab self‐collection and cervical cancer in women who have sex with women. Med J Aust. 2020;213(5):239‐239.e1. 10.5694/mja2.50736 32794591

[16] Smith MA , Sherrah M , Sultana F , et al. National experience in the first two years of primary human papillomavirus (HPV) cervical screening in an HPV vaccinated population in Australia: observational study. BMJ. 2022;376:e068582. 35354610 10.1136/bmj-2021-068582 PMC8965648

[17] Cancer Council Australia Self‐collected vaginal samples [Internet] . Cancer Council Australia. 2022. Available from: https://www.cancer.org.au/clinical‐guidelines/cervical‐cancer‐screening/management‐of‐oncogenic‐hpv‐test‐results/self‐collected‐vaginal‐samples

[18] Medical Services Advisory Commitee . Application No. 1664—Improvements to the National Cervical Screening Program Self‐Collection Policy, 2023. Medical Services Advisory Commitee. http://www.msac.gov.au/internet/msac/publishing.nsf/Content/69F7A5B132EA653ECA258646001B5CD5/$File/1664%20Final%20PSD%20‐%20Mar‐Apr%202021.pdf

[19] Foo Yun M , Goswami P , Grogin J , et al. Incorporation of human papillomavirus self‐sampling into the revised National Cervical Screening Program: a qualitative study of GP experiences and attitudes in rural New South Wales. Aust J Prim Health. 2021;27(4):284‐290. 33985644 10.1071/PY20209

[20] Obermair HM , Bennett KF , Brotherton JML , Smith MA , McCaffery KJ , Dodd RH . Australian National Cervical Screening Program renewal: attitudes and experiences of general practitioners, and obstetricians and gynaecologists. Aust N Z J Obstet Gynaecol. 2021;61(3):416‐423. 33512715 10.1111/ajo.13310

[21] Verbunt E , Boyd L , Creagh N , et al. Health care system factors influencing primary healthcare workers' engagement in national cancer screening programs: a qualitative study. Aust N Z J Public Health. 2022;46(6):858‐864. 35735902 10.1111/1753-6405.13272

[22] Sultana F , Roeske L , Malloy MJ , McDermott TL , Saville M , Brotherton JML . Implementation of Australia's renewed cervical screening program: preparedness of general practitioners and nurses. PLoS One. 2020;15(1):e0228042. 31995585 10.1371/journal.pone.0228042 PMC6988932

[23] Zammit CM , Creagh NS , McDermott T , et al. So, if she wasn't aware of it, then how would everybody else out there be aware of it?’—key stakeholder perspectives on the initial implementation of self‐collection in Australia's cervical screening program: a qualitative study. Int J Environ Res Public Health. 2022;19(23):15776. 36497850 10.3390/ijerph192315776 PMC9739016

[24] Zammit C , Creagh NS , Nightingale C , et al. “I'm a bit of a champion for it actually” – a qualitative study of the experience of practitioner‐supported self‐collection for cervical screening among Victorian practitioners in the Australian National Cervical Screening Program. Prim Health Care Res Dev. 2023;24:E31. 37185205 10.1017/S1463423623000191 PMC10156465

[25] Jaenke R , Butler TL , Condon J , et al. Health care provider perspectives on cervical screening for aboriginal and Torres Strait islander women: a qualitative study. Aust N Z J Public Health. 2021;45(2):150‐157. 33683744 10.1111/1753-6405.13084

[26] Gertig D , Lee J . Supporting health care providers in cancer screening: the role of the National Cancer Screening Register. Med J Aust. 2023;219(3):94‐98. 37454360 10.5694/mja2.52029

[27] Holt DT , Helfrich CD , Hall CG , Weiner BJ . Are you ready? How health professionals can comprehensively conceptualize readiness for change. J Gen Intern Med. 2010;25(1):50‐55. 20077152 10.1007/s11606-009-1112-8 PMC2806967

[28] Damschroder LJ , Aron DC , Keith RE , Kirsh SR , Alexander JA , Lowery JC . Fostering implementation of health services research findings into practice: a consolidated framework for advancing implementation science. Implement Sci. 2009;7(4):50. 10.1186/1748-5908-4-50 PMC2736161 19664226

[29] Rogers EM . Diffusion of Innovations, 5th Edition. Free Press; 2003. Available from: http://ebookcentral.proquest.com/lib/unimelb/detail.action?docID=4935198

[30] Australian Institute of Health and Welfare . National Cervical Screening Program Monitoring Report 2023. AIHW, Australian Government; 2023. Available from: https://www.aihw.gov.au/reports/cancer‐screening/ncsp‐monitoring‐2023/summary

[31] Moxham R , Moylan P , Duniec L , et al. Knowledge, attitudes, beliefs, intentions and behaviours of Australian indigenous women from NSW in response to the National Cervical Screening Program changes: a qualitative study. Lancet Reg Health West Pac. 2021;13:100195. 34527986 10.1016/j.lanwpc.2021.100195 PMC8403896

[32] Williams D , Davies M , Fiander A , Farewell D , Hillier S , Brain K . Women's perspectives on human papillomavirus self‐sampling in the context of the UK cervical screening programme. Health Expect. 2017;20(5):1031‐1040. 28186384 10.1111/hex.12544 PMC5600225

[33] Hawkes D , Keung MHT , Huang Y , et al. Self‐collection for cervical screening programs: from research to reality. Cancer. 2020;12(4):1053. 10.3390/cancers12041053 PMC7226191 32344565

[34] Guirguis‐Blake JM , Henderson JT , Perdue LA . Periodic screening pelvic examination: evidence report and systematic review for the US preventive services task force. JAMA. 2017;317(9):954‐966. 28267861 10.1001/jama.2016.12819 PMC13502370

[35] Tonelli M , Gorber SC , Moore A , Thombs BD . Recommendations on routine screening pelvic examination: Canadian task force on preventive health care adoption of the American College of Physicians guideline. Can Fam Physician. 2016;62(3):211‐214. 26975912 PMC4984604

[36] Qaseem A , Humphrey LL , Harris R , Starkey M , Denberg TD . Screening pelvic examination in adult women: a clinical practice guideline from the American College of Physicians. Ann Intern Med. 2014;161(1):67‐72. 24979451 10.7326/M14-0701

[37] Gupta DM , Boland RJ , Aron DC . The physician's experience of changing clinical practice: a struggle to unlearn. Implement Sci. 2017;12(1):28. 28245849 10.1186/s13012-017-0555-2 PMC5331724

[38] Côté‐Boileau É , Denis JL , Callery B , Sabean M . The unpredictable journeys of spreading, sustaining and scaling healthcare innovations: a scoping review. Health Res Policy Syst. 2019;17(1):84. 31519185 10.1186/s12961-019-0482-6 PMC6744644

[39] Greenhalgh T , Papoutsi C . Spreading and scaling up innovation and improvement. BMJ. 2019;365:l2068. 31076440 10.1136/bmj.l2068 PMC6519511

[40] Serrano B , Ibáñez R , Robles C , Peremiquel‐Trillas P , de Sanjosé S , Bruni L . Worldwide use of HPV self‐sampling for cervical cancer screening. Prev Med. 2022;154:106900. 34861338 10.1016/j.ypmed.2021.106900

[41] Lim AW , Hollingworth A , Kalwij S , Curran G , Sasieni P . Offering self‐sampling to cervical screening non‐attenders in primary care. J Med Screen. 2017;24(1):43‐49. 27235844 10.1177/0969141316639346

[42] Bansil P , Wittet S , Lim J , Winkler J , Paul P , Jeronimo J . Acceptability of self‐collection sampling for HPV‐DNA testing in low‐resource settings: a mixed methods approach. BMC Public Health. 2014;14:596. 24927941 10.1186/1471-2458-14-596 PMC4061776

[43] Teng FF , Mitchell SM , Sekikubo M , et al. Understanding the role of embarrassment in gynaecological screening: a qualitative study from the ASPIRE cervical cancer screening project in Uganda. BMJ Open. 2014;4(4):e004783. 10.1136/bmjopen-2014-004783 PMC3987737 24727360

[44] Xiong S , Lazovich DA , Hassan F , et al. Health care personnel's perspectives on human papillomavirus (HPV) self‐sampling for cervical cancer screening: a pre‐implementation, qualitative study. Imp Sci Comms. 2022;3(1):130. 10.1186/s43058-022-00382-3 PMC9745769 36514133

[45] Zehbe I , Wakewich P , King AD , Morrisseau K , Tuck C . Self‐administered versus provider‐directed sampling in the Anishinaabek cervical cancer screening study (ACCSS): a qualitative investigation with Canadian first nations women. BMJ Open. 2017;7(8):e017384. 10.1136/bmjopen-2017-017384 PMC5588934 28864487

[46] Zelli J , Hum S , Lofters A , Dunn S . Clinician acceptability of self‐collected human papillomavirus swabs as a primary cervical cancer screening method. Col Fam Physicians Can. 2022;68(2):e31‐e38. 10.46747/cfp.6802e31 PMC9842175 35177513

[47] Rodriguez NM , Brennan LP , Claure L , Balian LN , Champion VL , Forman MR . Leveraging COVID‐era innovation for cervical cancer screening: clinician awareness and attitudes toward self‐sampling and rapid testing for HPV detection. PLoS One. 2023;18(3):e0282853. 36893182 10.1371/journal.pone.0282853 PMC9997915

[48] Chua BWB , Neo P , Ma VY , Lim LM , Ng JSY , Wee HL . Health care provider's experience and perspective of cervical cancer screening in Singapore: a qualitative study. Public Health Front. 2022;10:853453. 10.3389/fpubh.2022.853453 PMC9360748 35958842

[49] Australian Government Department of Health and Aged Care . National Strategy for the Elimination of Cervical Cancer in Australia [Internet]. Australian Centre for the Prevention of Cervical Cancer; 2023. Available from: https://www.health.gov.au/resources/publications/national‐strategy‐for‐the‐elimination‐of‐cervical‐cancer‐in‐australia?language=en

